# The impact of circWWC3 on neoadjuvant therapy for triple-negative breast cancer and the construction of a nomogram for predicting pathological complete response after neoadjuvant therapy

**DOI:** 10.3389/fonc.2025.1564693

**Published:** 2025-07-11

**Authors:** Haoqi Wang, Hongbo Liu, Shan Gao, Lijia Wang, Zihao Bai, Yi Zhang, Peijin Zhang, Fei Liu, Cuizhi Geng

**Affiliations:** ^1^ Department of Breast Center, The Fourth Hospital of Hebei Medical University, Shijiazhuang, Hebei, China; ^2^ Hebei Key Laboratory of Breast Cancer Molecular Medicine, The Fourth Hospital of Hebei Medical University, Shijiazhuang, Hebei, China; ^3^ Gland Surgery, The Hebei Province People’s Hospital, Shijiazhuang, Hebei, China; ^4^ Department of Imaging, The Fourth Hospital of Hebei Medical University, Shijiazhuang, Hebei, China; ^5^ Research Center and Tumor Research Institute, The Fourth Hospital of Hebei Medical University, Shijiazhuang, Hebei, China

**Keywords:** triple-negative breast cancer, circWWC3, neoadjuvant therapy, pathological complete response, nomogram

## Abstract

**Background:**

The introduction of novel strategies for neoadjuvant therapy (NAT) has significantly enhanced the rate of pathological complete response (pCR) in patients with triple-negative breast cancer (TNBC). However, due to tumor heterogeneity, some patients continue to experience poor treatment efficacy, early recurrence, metastasis, and even mortality. Therefore, it is crucial to identify new molecular targets for precise treatment and to develop predictive models for pCR to facilitate tailored therapeutic approaches.

**Methods:**

We conducted a study involving a cohort of TNBC patients who underwent NAT, collecting data on clinicopathological indicators, MRI parameters, and pathological remission outcomes. The expression levels of baseline circular RNA WWC3 (circWWC3) in breast cancer tissue were assessed, and the relationship between its expression and clinicopathological indicators as well as pathological response was analyzed. A nomogram for predicting pCR in TNBC was developed and subsequently validated.

**Results:**

From January 2020 to December 2023, a total of 205 patients were included in the final analysis. The rate of total pathological complete response (tpCR), defined as ypT0/is and ypN0, was observed to be 51.7%. CircWWC3 was found to be highly expressed in the cytoplasm, with an expression rate of 79% among all analyzed cancerous tissues. The elevated expression of circWWC3 was positively correlated with T2 stage, N1 status, Ki-67 levels greater than 30%, and moderate to high infiltration of tumor-infiltrating lymphocytes (TILs) (*p* < 0.05). Additionally, a change rate in the apparent diffusion coefficient (ADC) of breast MRI after two cycles of neoadjuvant therapy (ΔADC _0-2_%) greater than 24.53% was significantly associated (*p* < 0.05). Patients exhibiting high levels of circWWC3 expression were more likely to achieve pCR. Univariate and multivariate regression analyses identified TILs, ΔADC _0-2_%, and circWWC3 as key variables for constructing a predictive nomogram for pCR. This model demonstrated strong discrimination, calibration, and clinical applicability.

**Conclusion:**

Elevated expression levels of circWWC3 serve as an independent risk factor influencing the likelihood of achieving a pCR. A predictive model that integrates circWWC3 expression alongside pathological and imaging parameters demonstrates a robust capacity to accurately forecast the probability of pCR in patients diagnosed with TNBC.

## Introduction

Triple-negative breast cancer (TNBC) is a subtype of breast cancer characterized by the absence of estrogen receptor (ER), progesterone receptor (PR), and human epidermal growth factor receptor 2 (HER2) expression. Although TNBC has the lowest incidence among breast cancer (BC) subtypes, it presents significant treatment challenges due to limited therapeutic options and its aggressive nature (1, 2). Neoadjuvant therapy (NAT) is commonly employed for patients with newly diagnosed breast cancer to facilitate tumor downstaging, enable breast-conserving surgery, and guide subsequent adjuvant therapy based on pathological response (3, 4). Evidence has established a correlation between complete pathological response (pCR) following NAT and improved survival outcomes in TNBC patients (5). However, the pCR rates achieved with chemotherapy alone remain suboptimal (6). While the incorporation of immunotherapy has been shown to enhance pCR rates (7), some patients still experience early disease recurrence and poor prognosis, attributed to the high heterogeneity of the tumor (8). Therefore, it is crucial to investigate new therapeutic targets for the treatment of TNBC.

Circular RNAs (circRNAs), which are noncoding RNAs generated from precursor (pre)-mRNA through back splicing, are characterized by their covalently closed structure and enhanced stability compared to linear RNAs (9, 10). Evidence suggests that circRNAs play significant roles in tumorigenesis and progression, functioning as either oncogenes or tumor suppressors through various mechanisms (11). Our previous research demonstrated that Zinc finger E-box binding homeobox 1 (ZEB1) upregulates circular RNA WWC3 (circWWC3), thereby promoting BC progression via the activation of the Ras signaling pathway (12). Furthermore, circWWC3 may facilitate BC progression by inducing M2 macrophage polarization and tumor immune evasion through the upregulation of interleukin-4 (IL-4) expression and secretion (13). Elevated levels of circWWC3 have been associated with poor disease-free survival (DFS) (13) and overall survival (OS) (12, 13). However, the role of circWWC3 in the context of NAT for TNBC remains unexplored.

In this study, we examined the expression of circWWC3 in patients with newly diagnosed TNBC and explored its association with various clinicopathological parameters, as well as its relationship with pCR following NAT. Additionally, we developed a nomogram to predict the likelihood of achieving pCR in TNBC patients. Our findings provided new therapeutic targets for TNBC, laid the foundation for earlier assessment of the efficacy of NAT for TNBC, and further provided a basis for individualized therapy.

## Materials and methods

### Patients

Patients who received NAT at the Fourth Hospital of Hebei Medical University from January 2020 to December 2023 were included in this study. The inclusion criteria were as follows: 1) Female, 2) Newly diagnosed and previously untreated TNBC, 3) Unilateral breast cancer without evidence of metastasis to internal mammary or supraclavicular lymph nodes, 4) Completion of the full course of NAT followed by radical breast surgery, 5) Regular breast MRI examinations conducted prior to and during NAT (every two cycles) and before surgery, 6) Availability of comprehensive clinicopathological and follow-up data. The exclusion criteria included: 1) Bilateral or occult breast cancer, 2) A history of other malignancies, 3) Insufficient clinicopathological data, 4) Loss to follow-up.

This study was approved by the ethics committee of the Fourth Hospital of Hebei Medical University. No identifiable or private patient information was involved; furthermore, as this study did not involve any interventions, the requirement for informed consent was waived.

### Tumor specimens

All cancerous specimens were obtained through puncture biopsy from patients diagnosed with TNBC. The samples were subsequently preserved and embedded in paraffin blocks, which were then sectioned into slides for the detection of circWWC3.

### Chromogenic *in situ* hybridization

Slides were dewaxed using xylene and ethanol solutions of varying concentrations. Following a prehybridization step at 37°C for one hour, the slides were hybridized overnight at 37°C with probes targeting circWWC3. The expression of circWWC3 was assessed using a 3, 3’-diaminobenzidine (DAB) color development agent, and the nuclei were counterstained with hematoxylin. Images were captured at different magnifications. The expression of circWWC3 was evaluated based on the intensity of cytoplasmic staining and the percentage of stained tumor cells (14). Moderate to strong staining intensity observed in the majority of cells was classified as high expression, while all other cases were categorized as low expression.

### Clinicopathologic information and variable definitions

Clinicopathologic data were collected from eligible patients, including age, menstrual status, tumor size, axillary lymph node staging, TNM stage, hormone receptor (HR) status, HER2 expression, Ki-67 index, infiltration status of tumor-infiltrating lymphocytes (TILs), change rate in the apparent diffusion coefficient (ADC) of breast MRI after two cycles of neoadjuvant therapy (ΔADC _0-2_%), NAT regimens, surgical methods, Miller-Payne (MP) classification (15), and the condition of residual cancer burden (RCB). The ER, PR, HER2, and Ki-67 were assessed using immunohistochemical (IHC) staining. HR-negative status was defined as ER and PR expression of less than 1% (16). Given the similar biological behavior of negative and weak HR expression (ER and PR: 1 - 10%), TNBC was defined as HR expression of 0-10% along with HER2 negativity. The infiltration status of TILs was evaluated through hematoxylin-eosin (HE) staining and categorized as low (0% - 10%), moderate (11% - 59%), and high (> 60%) (17). The ΔADC _0-2_% was calculated using the formula: (ADC value after two cycles of NAT - ADC value at baseline)/ADC value at baseline × 100%. Based on our previous research, ΔADC _0-2_% was classified into two groups: ΔADC _0-2_% > 24.53% and ΔADC _0-2_% ≤ 24.53% (18).

### Assessment and endpoints

Imaging assessments of the breast and the lymph nodes within the drainage area were conducted at baseline, every two cycles of NAT, and prior to surgery, utilizing breast-enhanced magnetic resonance imaging (MRI) and ultrasound. The pathological response was evaluated according to the MP grading system and the RCB scoring system. The primary endpoint of the study was the rate of total pCR (tpCR, defined as ypT0/is and ypN0). The secondary endpoint was the rate of breast pathological complete response (bpCR, defined as ypT0/is).

### Statistical analysis

Data analysis was conducted using SPSS version 27.0, with categorical data presented as n (%). The Chi-square test was employed to assess differences between the two groups. Multivariate binary logistic regression analysis was performed to identify independent predictors of pCR. A nomogram for predicting pCR was developed using the ‘rms’ package in R software, based on the identified independent predictors. The Receiver Operating Characteristic (ROC) curve was generated using the ‘proc’ and ‘ggplot2’ packages to calculate the area under the curve (AUC), as well as to determine the optimal cutoff value, specificity, and sensitivity. The accuracy of the model was evaluated using the Hosmer-Lemeshow Calibration Curve. Additionally, Decision Curve Analysis (DCA) was performed using the ‘rmda’ package to assess the clinical applicability of the model. Internal validation of the model was conducted through the Bootstrap self-sampling method. A p-value of less than 0.05 was considered statistically significant.

## Results

### Demographic characteristics

A total of 469 patients with TNBC received NAT between January 2020 and December 2023, of which 205 patients met the inclusion criteria for the final analysis. Patient demographics are summarized in **Table 1**. The majority of patients were over 50 years of age and postmenopausal. At initial diagnosis, 62.5% of patients presented with T2 tumors. Only 15.1% of patients exhibited no lymph node metastasis, while the majority were found to have lymph node involvement. All patients were staged at II-III, with a similar distribution between the two stages. Negative ER was observed in 92.2% of patients. The incidence of HER2 ‘0 expression’ was comparable to that of HER2 ‘low expression’. High Ki-67 expression (greater than 30%) was noted in 68.8% of patients, and the infiltration of TILs was predominantly low (50.7%) and moderate (40.0%). The proportions of patients with ΔADC _0-2_% greater than 24.53% (52.7%) and those with ΔADC _0-2_% less than or equal to 24.53% (47.3%) were comparable. Among the NAT regimens administered, those containing anthracyclines were the most prevalent (53.2%), followed by regimens incorporating platinum drugs (34.6%), anthracycline/platinum sequential regimens, and taxane monotherapy. A minority of patients (25.3%) received combined therapies, including immunotherapy or antiangiogenic agents.

**Table 1:** Demographic and clinicopathological parameters of patients with triple-negative breast cancer.

Clinicopathological indicators	N, n(%)
Age, n (%)	
> 50	131 (63.9%)
≤ 50	74 (36.1%)
Menstrual status, n (%)	
postmenopausal	115 (56.1%)
premenopausal	90 (43.9%)
T stage, n (%)	
T1	23 (11.2%)
T2	128 (62.5%)
T3	31 (15.1%)
T4	23 (11.2%)
N stage, n (%)	
N0	31 (15.1%)
N1	108 (52.7%)
N2	38 (18.5%)
N3	28 (13.7%)
TNM stage, n (%)	
IIA	33 (16.1%)
IIB	79 (38.5%)
IIIA	46 (22.4%)
IIIB	19 (9.3%)
IIIC	28 (13.7%)
HR ^a^, n (%)	
0%	189 (92.2%)
1-10%	16 (7.8%)
HER2, n (%)	
0-expression	108 (52.7%)
Low-expression	97 (47.3%)
Ki-67, n (%)	
> 30%	141 (68.8%)
≤ 30%	64 (31.2%)
TILs^b^ infiltration, n (%)	
Low-infiltrated	104 (50.7%)
Moderate-infiltrated	82 (40.0%)
High-infiltrated	19 (9.3%)
ΔADC _0-2_% ^c^, n (%)	
≤ 24.53%	97 (47.3%)
> 24.53%	108 (52.7%)
Chemotherapy regimen, n (%)	
platinum	71 (34.6%)
anthracyclic	109 (53.2%)
platinum and anthracyclic sequential	18 (8.8%)
taxane monotherapy	7 (3.4%)
Combined therapy, n (%)	
immunotherapy	23 (11.2%)
antiangiogenic drugs	29 (14.1%)
none	153 (74.7%)
Surgery, n (%)	
complete mastectomy	142 (69.3%)
breast-conserving surgery	63 (30.7%)

^a^HR, hormone receptor; ^b^TILs, tumor-infiltrating lymphocytes; ^c^△ADC _0-2_%, change rate of ADC after two cycles of neoadjuvant therapy

### Efficacy

All patients underwent radical mastectomy following a complete course of NAT. Among these patients, 30.7% underwent breast-conserving surgery (BCS), while 69.3% received total mastectomy. A total of 106 patients achieved a tpCR following NAT, resulting in an overall pCR rate of 51.7%. Additionally, 57.6% of the patients attained a bpCR. The details regarding the pathological remission outcomes for the patients are presented in **Table 2**.

**Table 2:** Pathological remission of patients through neoadjuvant therapy.

MP^a^	N (%)	RCB^b^	N (%)
1	7 (3.4%)	0	106 (51.7%)
2	8 (3.9%)	1	23 (11.2%)
3	43 (21.0%)	2	53 (25.9%)
4	29 (14.1%)	3	23 (11.2%)
5	118 (57.6%)		

^a^MP, Miller-Payne; ^b^RCB, residual cancer burden.

### Expression of circWWC3 in TNBC tissue and its effect on the efficacy of NAT

**Figure 1:**
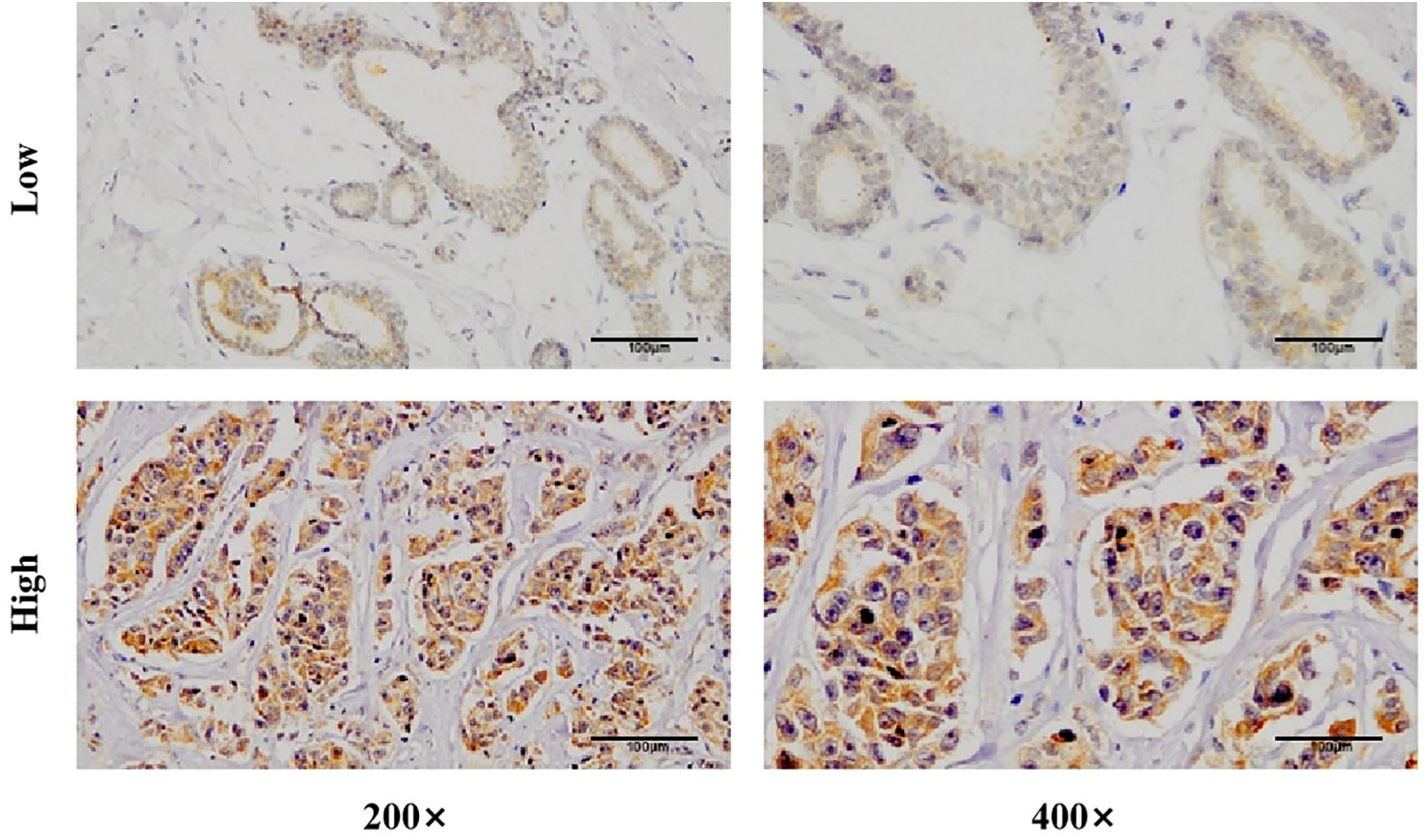
The decision curve analysis (DCA) curve for the nomogram.

CISH detection revealed that circWWC3 was predominantly localized in the cytoplasm. Among the analyzed samples, 162 out of 205 cases (79.0%) exhibited high levels of circWWC3 expression, while 43 cases (21.0%) demonstrated low expression levels. A representative image illustrating the varying levels of circWWC3 expression as assessed by CISH is presented in **Figure 1**. Following NAT, a tpCR was observed in 63.5% (103/162) of patients exhibiting high circWWC3 expression. In contrast only 3 patients (3/43, 6.9%) with low circWWC3 expression achieved tpCR (*p* < 0.001). High levels of circWWC3 expression were predominantly identified in patients classified as MP 4–5 and RCB 0-I, whereas low circWWC3 expression was more frequently observed in patients categorized as MP 1–3 and RCB II-III (*p* < 0.001) (**Table 3**).

**Table 3:** Relationship between circWWC3 expression and pathological remission after neoadjuvant therapy.

MP ^a^	circWWC3 expression		*p*	RCB ^b^	circWWC3 expression		*p*
	H^c^ (162)	L^d^ (43)			H^c^ (162)	L^d^ (43)	
1-3	18 (11.1%)	40 (93.0%)	<0.001	0-1	126 (77.8%)	3 (7.0%)	<0.001
4-5	144 (88.9%)	3 (7.0%)		2-3	36 (22.2%)	40 (93.0%)	

^a^MP, Miller-Payne; ^b^RCB, residual cancer burden; ^c^H, High expression; ^d^L, Low expression.

### Associations between circWWC3 expression and clinicopathological parameters

As presented in **Table 4**, the expression levels of circWWC3 demonstrated a significant correlation with tumor size, lymph node metastasis status, Ki-67 expression, TILs infiltration, and ΔADC _0-2_% (*p* < 0.05). High expression of circWWC3 were predominantly observed in patients classified as T2N1, exhibiting elevated Ki-67 expression, and demonstrating moderate to high levels of TILs. Additionally, a ΔADC _0-2_% greater than 24.53% was associated with increased circWWC3 expression. However, circWWC3 expression did not show a significant correlation with other clinical pathological parameters (*p* > 0.05).

**Table 4:** Relationship between circWWC3 expression and clinicopathological parameters.

Clinicopathological indicators	circWWC3 expression		*P*
	High (162)	Low (43)	
Age, n (%)			0.864
> 50	104 (64.2%)	27 (62.8%)	
≤ 50	58 (35.8%)	16 (37.2%)	
Menstrual status, n (%)			0.320
postmenopausal	88 (54.3%)	27 (62.8%)	
premenopausal	74 (45.7%)	16 (37.2%)	
T stage, n (%)			0.049
T1	21 (13.0%)	2 (4.6%)	
T2	105 (64.8%)	23 (53.5%)	
T3	20 (12.3%)	11 (25.6%)	
T4	16 (9.9%)	7 (16.3%)	
N stage, n (%)			0.019
N0	21 (13.0%)	10 (23.3%)	
N1	91(56.2%)	17 (39.5%)	
N2	25 (15.4%)	13 (30.2%)	
N3	25 (15.4%)	3 (7.0%)	
TNM stage, n (%)			0.099
IIA	27 (16.7%)	6 (14.0%)	
IIB	66 (40.8%)	13 (30.2%)	
IIIA	31 (19.1%)	15 (34.8%)	
IIIB	13 (8.0%)	6 (14.0%)	
IIIC	25 (15.4%)	3 (7.0%)	
HR^a^, n (%)			0.927
0%	150 (92.6%)	39 (90.7%)	
1-10%	12 (7.4%)	4 (9.3%)	
HER2, n (%)			0.644
0-expression	84 (51.9%)	24 (55.8%)	
Low-expression	78 (48.1%)	19 (44.2%)	
Ki-67, n (%)			<0.001
> 30%	111 (68.5%)	13 (30.0%)	
≤ 30%	51 (31.5%)	30 (70.0%)	
TILs ^b^infiltration, n (%)			<0.001
Low-infiltrated	70 (43.2%)	34 (79.1%)	
Moderate and high-infiltrated	92 (56.8%)	9 (20.9%)	
ΔADC _0-2_% ^c^, n (%)			<0.001
≤ 24.53%	56 (34.6%)	41 (95.3%)	
> 24.53%	106 (65.4%)	2 (4.7%)	

^a^HR, hormone receptor; ^b^TILs, tumor-infiltrating lymphocytes; ^c^△ADC _0-2_%, change rate of ADC after two cycles of neoadjuvant therapy.

### Clinicopathological indicators affecting pCR

Patients in the premenopausal state with highly infiltrated TILs, and ΔADC _0-2_% greater than 24.53% were more likely to achieve a tpCR (*p* < 0.001). Additionally, the rate of tpCR was higher among individuals exhibiting low HER2 expression, those treated with platinum-containing regimens or combination therapies, and patients with high Ki-67 expression, in spite of no statistical difference (*p* > 0.05) (**Table 5**).

**Table 5:** Relationship between clinicopathological parameters and pathological complete response.

Clinicopathological indicators	pCR ^a^ (106)	non-pCR^a^ (99)	*P*
Age, n (%)			0.277
> 50	64 (60.4%)	67 (67.7%)	
≤ 50	42 (39.6%)	32 (32.3%)	
Menstrual status, n (%)			0.036
postmenopausal	52 (49.1%)	63 (63.6%)	
premenopausal	54 (50.9%)	36 (36.4%)	
T stage, n (%)			0.579
T1	15 (14.1%)	8 (8.1%)	
T2	64 (60.4%)	64 (64.6%)	
T3	16 (15.1%)	15 (15.2%)	
T4	11(10.4%)	12 (12.1%)	
N stage, n (%)			0.945
N0	16 (15.1%)	15 (15.2%)	
N1	57 (53.8%)	51 (51.5%)	
N2	18 (17.0%)	20 (20.2%)	
N3	15 (14.1%)	13 (13.1%)	
TNM stage, n (%)			0.980
IIA	18 (17.0%)	15 (15.2%)	
IIB	41 (38.7%)	38 (38.4%)	
IIIA	22 (20.8%)	24 (24.2%)	
IIIB	10 (9.4%)	9 (9.1%)	
IIIC	15 (14.1%)	13 (13.1%)	
HR ^b^, n (%)			0.155
0%	95 (89.6%)	94 (94.9%)	
1-10%	11 (10.4%)	5 (5.1%)	
HER2, n (%)			0.426
0-expression	53 (50.0%)	55 (55.6%)	
Low-expression	53 (50.0%)	44 (44.4%)	
Ki-67, n (%)			0.125
> 30%	78 (73.6%)	63(63.6%)	
≤ 30%	28 (26.4%)	36 (36.4%)	
TILs^c^ infiltration, n (%)			<0.001
Low-infiltrated	35 (33.0%)	69 (69.7%)	
Moderate-infiltrated	54 (50.9%)	28 (28.3%)	
High-infiltrated	17 (16.1%)	2 (2.0%)	
ΔADC _0-2_% ^d^, n (%)			<0.001
≤ 24.53%	29 (27.4%)	68 (68.7%)	
> 24.53%	77 (72.6%)	31 (31.3%)	
Chemotherapy regimen, n (%)			0.103
platinum	44 (41.5%)	27 (27.3%)	
anthracyclic	53 (50.0%)	56 (56.5%)	
platinum and anthracyclic sequential	7 (6.6%)	11 (11.1%)	
taxane monotherapy	2 (1.9%)	5 (5.1%)	
Combined therapy, n (%)			0.259
immunotherapy	14 (13.2%)	9 (9.1%)	
antiangiogenic drugs	18 (17.0%)	11 (11.1%)	
none	74 (69.8%)	79 (79.8%)	

^a^pCR, pathological complete response; ^b^HR, hormone receptor; ^c^TILs, tumor-infiltrating lymphocytes; ^d^△ADC _0-2_%, change rate of ADC after two cycles of neoadjuvant therapy.

### Multivariate binary logistic regression analysis for affecting pCR

Further multivariate analysis revealed that TILs infiltration was an independent predictor of tpCR, with odds ratios (OR) of 2.663 (95% confidence interval [CI] = 1.337 - 5.301) for moderate infiltration and 15.015 (95% CI = 2.813 - 80.148) for high infiltration. Additionally, ΔADC _0-2_% was associated with an OR of 3.131 (95% CI = 1.541 - 6.364). Furthermore, circWWC3 expression emerged as a significant independent factor, with an OR of 9.827 (95% CI = 2.636 - 36.632) for predicting tpCR (**Table 6**).

**Table 6:** Multivariate binary Logistic regression analysis affecting pathological complete response.

Variables	B	S.E	Wald	P	OR	95%CI
TILs^a^						
Low			15.035	0.001		
Moderate	0.979	0.351	7.767	0.005	2.663	1.337-5.301
High	2.709	0.855	10.051	0.002	15.015	2.813-80.148
ΔADC _0-2_%^b^	1.141	0.362	9.952	0.002	3.131	1.541-6.364
circWWC3 expression	2.285	0.671	11.586	0.001	9.827	2.636-36.632
Constant	-5.374	1.269	17.929	0.000	.005	

^a^TILs, tumor-infiltrating lymphocytes; ^b^△ADC _0-2_%, change rate of ADC after two cycles of neoadjuvant therapy.

### Construction of a nomogram for predicting pCR after NAT for TNBC

**Figure 2:**
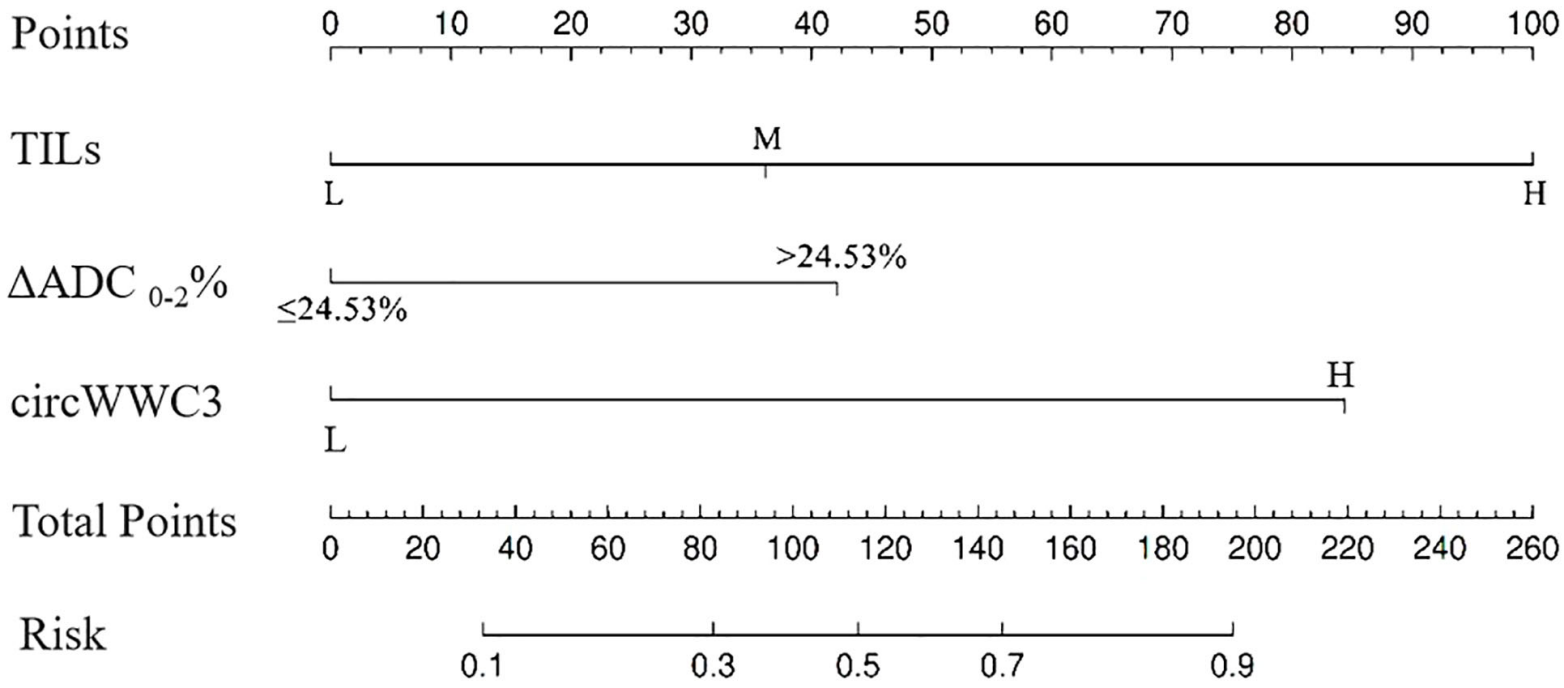
The nomogram for predicting pathological complete response after neoadjuvant therapy in patients with triple negative breast cancer.

Based on the aforementioned analysis, the TILs, ΔADC _0-2_%, and the expression levels of circWWC3 were utilized to construct a nomogram for predicting pCR following NAT (**Figure 2**). The points assigned to each predictor correspond to their respective scores for tpCR. Among the predictors evaluated, a high level of TIL infiltration emerged as the most significant indicator of pCR. The cumulative scores for each predictor were summed, with the resulting risk value at the bottom reflecting the likelihood of achieving tpCR.

### Discrimination analysis of the nomogram model

**Figure 3:**
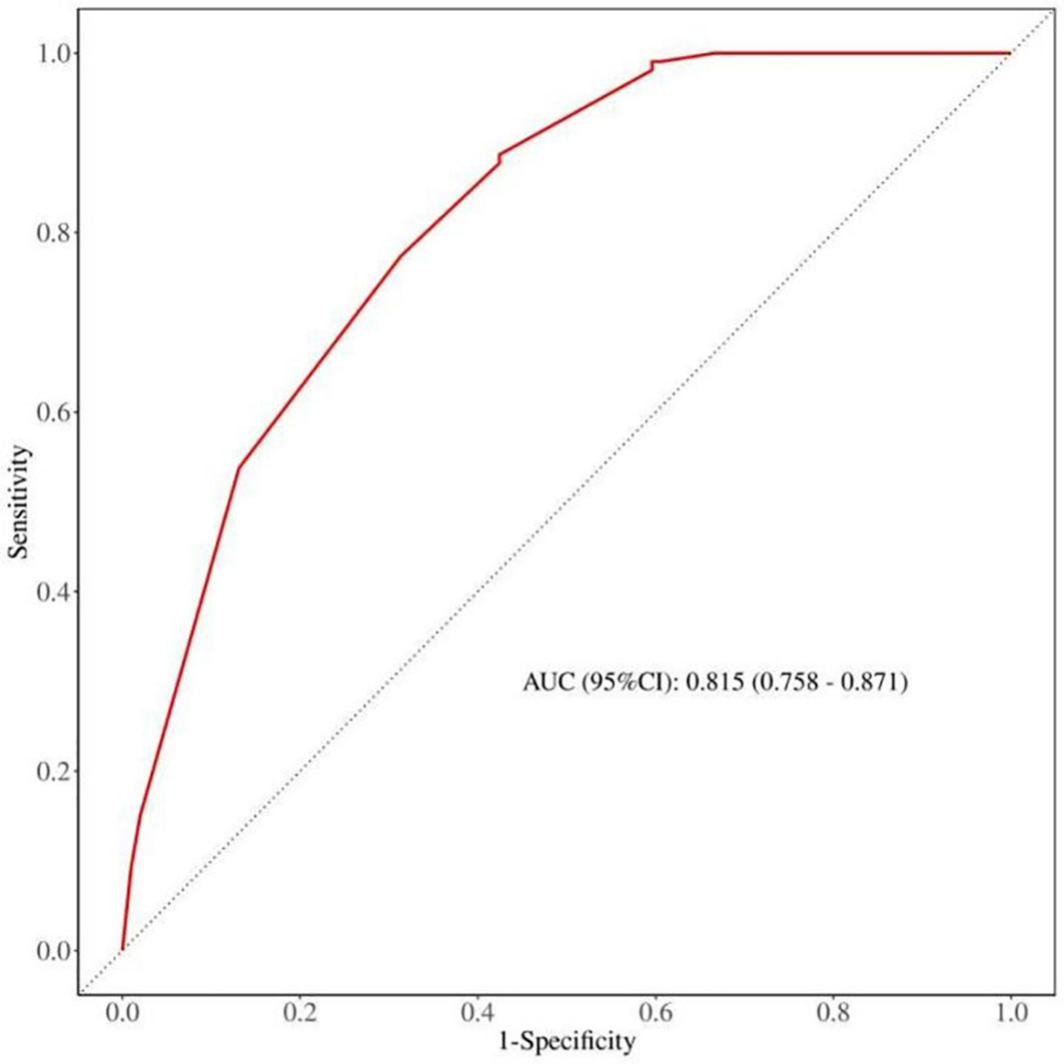
The expression of circWWC3 in triple negative breast cancer tissues by chromogenic*in situ* hybridization (CISH).

The discrimination capability of the nomogram model was assessed using ROC curve analysis, yielding an AUC value of 0.815 (95% CI: 0.758 - 0.871; *p* < 0.001). At the optimal threshold of 0.358, the model demonstrated a sensitivity of 0.567 and a specificity of 0.877. Additionally, internal validation was conducted through bootstrap resampling, performed 1,000 times, which resulted in an AUC value of 0.821, further corroborating the model’s robust discriminatory performance (**Figure 3**).

### Calibration analysis of nomogram model

**Figure 4:**
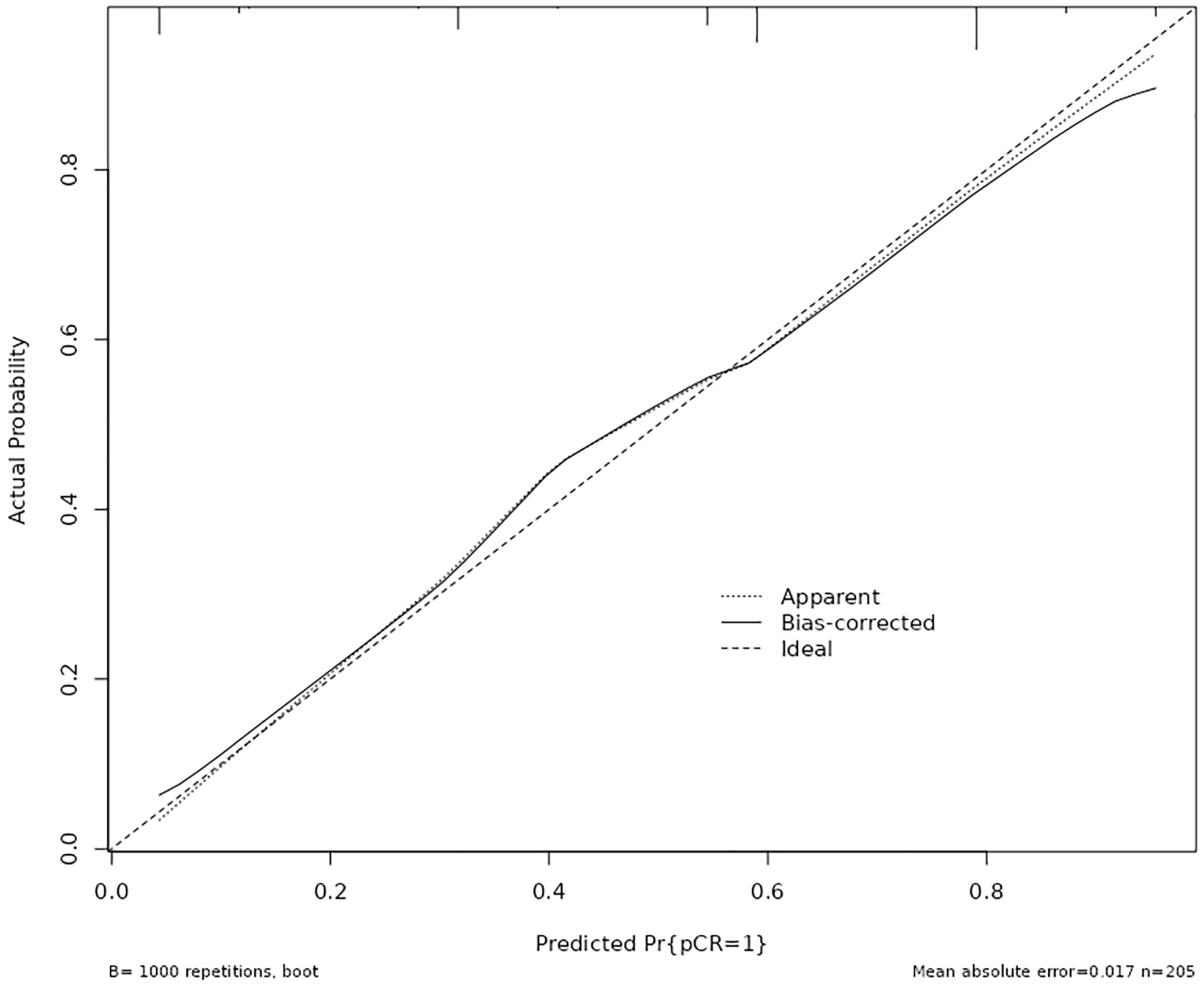
Calibration plots for the nomogram to predict pathological complete response after neoadjuvant therapy.

The calibration curve was employed to assess the calibration performance of the nomogram. The results indicated no significant difference between the predicted and actual probabilities (χ² = 2.118, df = 4, *p* = 0.714). Following internal validation via Bootstrap repeated sampling, the absolute error was determined to be 0.017. As illustrated in **Figure 4**, the calibration curve closely approximated the ideal curve, with the original curve also showing a slope near 1, indicating a strong concordance between the actual and predicted probabilities of pCR occurrence.

### Evaluation for clinical practicability of nomogram model

**Figure 5:**
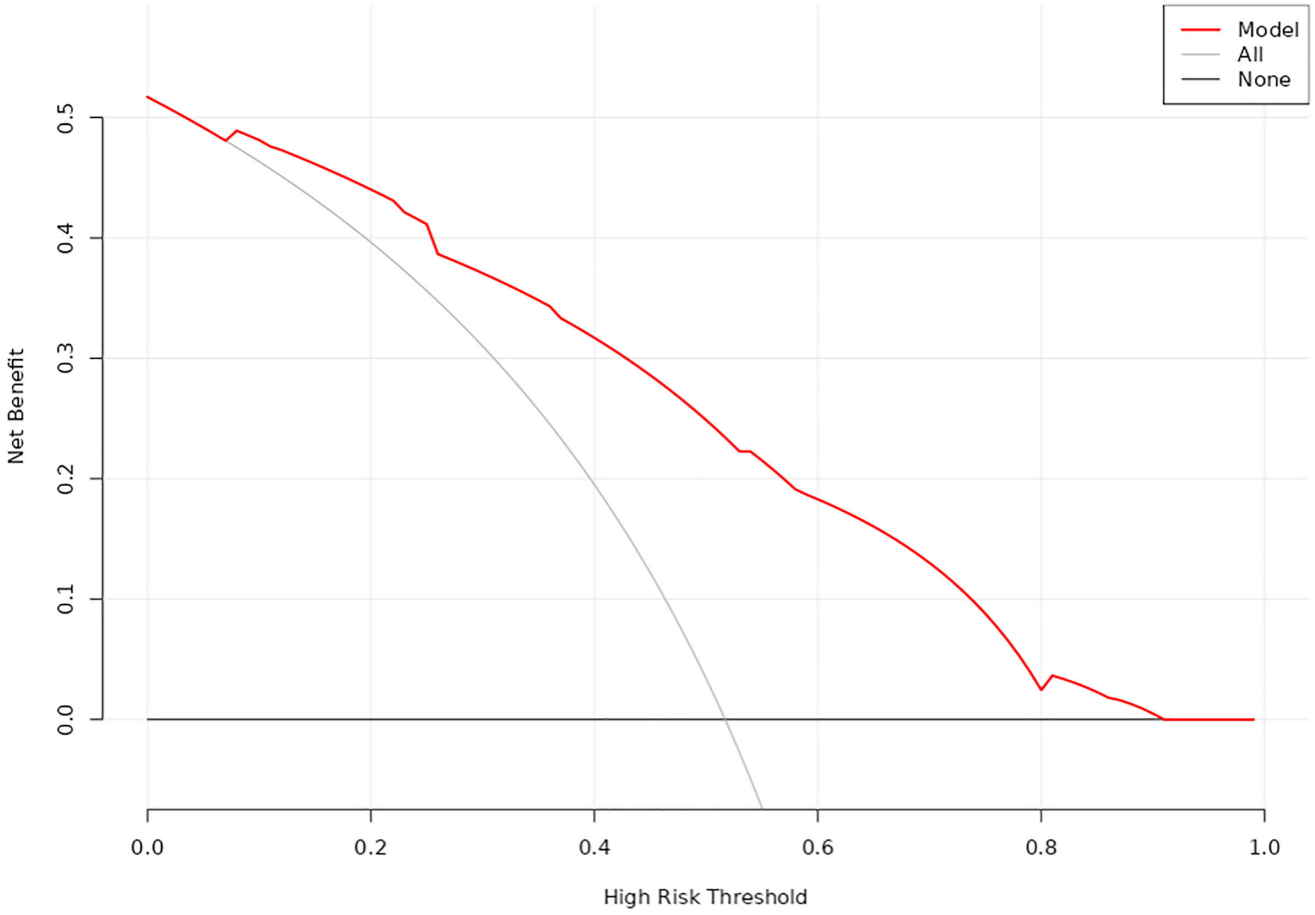
The receiver operating characteristic (ROC) curve for the nomogram validation.

DCA was utilized to evaluate the clinical utility of the nomogram model (**Figure 5**). The analysis revealed that when the threshold probability ranged from 7% to 92%, the application of this model yielded a higher clinical net benefit, significantly surpassing the net benefit levels of the None line and All line. This finding underscores the model’s substantial clinical applicability.

## Discussion

TNBC remains one of the most challenging subtypes to treat due to its high heterogeneity, aggressiveness, and limited therapeutic options. Consequently, precision typing and tailored treatment strategies are essential in contemporary clinical practice. Jiang et al. (19) classified TNBC into four distinct subtypes by analyzing clinical, genomic, and transcriptomic data from 465 patients diagnosed with primary TNBC. These subtypes include luminal androgen receptor (LAR), immunomodulatory, basal-like immune-suppressed, and mesenchymal-like. Based on the identified potential therapeutic targets and biomarkers for each subtype, several clinical trials have been initiated, with preliminary results reported (20–22). While the refinement of treatment protocols informed by precision typing has led to a modest improvement in the survival of TNBC patients, these advancements primarily focus on patients in the advanced stage, with limited studies addressing early-stage or locally advanced patients. Achieving a cure for early locally advanced breast cancer is critical for enhancing overall prognosis. Given the correlation between pCR and long-term survival, the current standard of care in NAT for TNBC involves chemotherapy combined with anti-PD-1 immunotherapy (23). However, some patients continue to exhibit suboptimal responses and may experience immune-related adverse events (7, 8). Therefore, exploring new therapeutic targets within the context of NAT to facilitate precision typing and therapy holds significant clinical value.

CircWWC3 has been established as a factor implicated in tumor progression, albeit with limited conflicting evidence documented in the literature. Liu et al. (24) demonstrated that circWWC3 inhibits cell growth, migration, and invasion while promoting apoptosis in osteosarcoma through the regulation of the miR-421/PDE7B axis, thereby reducing tumorigenicity in murine xenograft models. Conversely, circWWC3 is found to be highly expressed in renal carcinoma tissues compared to normal tissues, and its elevated expression correlates with poor prognosis in patients with renal carcinoma (14). In the context of breast cancer, our findings indicate that circWWC3 functions as an oncogene, enhancing tumor cell aggressiveness and influencing the tumor microenvironment (TME) (12, 13). Notably, high levels of circWWC3 are associated with unfavorable OS and DFS outcomes (13). To our knowledge, this study represents the first investigation into the impact of circWWC3 expression on the efficacy of NAT in patients with TNBC. Our results suggest that elevated circWWC3 levels are significantly associated with tumors classified as T2 or N1, likely due to the relatively high proportion of such tumors. Additionally, high circWWC3 expression is more prevalent in cases exhibiting markers indicative of a propensity to achieve pCR, including elevated Ki-67 expression (25), moderate to high levels of TILs (26), and ΔADC _0-2_% > 24.53% (18). Furthermore, it has been confirmed that patients with high circWWC3 expression are more likely to attain tpCR. Therefore, exploring new therapeutic targets in the context of NAT holds significant clinical value for achieving precision typing and therapy.

A series of studies have sought to develop predictive models for pCR following NAT in TNBC by utilizing various indicators. In addition to clinicopathological factors, most documented nomograms have incorporated radiomic signatures derived from imaging modalities such as ultrasound (27–29), contrast-enhanced computed tomography (CECT) (30, 31), and MRI (32, 33). Notably, only two predictive models have integrated laboratory indicators (34) or employed a novel biomarker-based predictive response score (pRS) system (35). In the MRI-based nomogram, parameters such as tumor volume measured via dynamic contrast-enhanced MRI (DCE-MRI), time to peak (TTP), and a radiomics score derived from contrast agent plasma-to-interstitial transfer (Ktrans), volume fraction of extravascular and extracellular space (Ve), and maximum contrast agent uptake rate (Slopemax) were included. These parameters were analyzed using unsupervised correlation and the least absolute shrinkage and selection operator (LASSO) methodology (30, 31). In our previous research, we established a predictive nomogram for pCR in patients with various breast cancer subtypes by incorporating the change rate of ADC in enhanced MRI after two cycles of NAT. This model demonstrated strong discrimination, calibration, and clinical applicability (18). According to our findings, TILs infiltration, circWWC3 expression prior to NAT, and ΔADC _0-2_% were identified as independent predictors of pCR. These factors were utilized to construct a prediction nomogram for pCR, which exhibited significant predictive power and clinical relevance. To our knowledge, this represents the first nomogram that predicts pCR by integrating pathological indicators, biomarkers, and imaging parameters.

Our study has several limitations. Firstly, the sample size was constrained, although we employed the bootstrap repeated sampling method for internal validation of the model, further large-scale validation is essential. Secondly, the model was developed using data from a single center, necessitating verification and application across multiple centers. Thirdly, the follow-up period was relatively short, and there was a lack of data on the correlation between circWWC3 expression and long-term survival. In the future, we will report on this topic. Nevertheless, our study still holds certain clinical value: we have, for the first time, reported that the positive expression of circWWC3 is associated with pCR following NAT for TNBC. Based on multi-dimensional indicators (circWWC3 and clinical pathological indicators), we have constructed an early prediction model for pCR after NAT for TNBC. This model can assist clinicians in assessing the efficacy of NAT earlier, thereby allowing for timely adjustment of treatment plans and avoiding ineffective treatments and potential adverse reactions.

## Conclusions

In conclusion, we found that high expression levels of circWWC3 in TNBC are significantly associated with an increased likelihood of achieving a pCR. An integrative predictive model for pCR incorporating pathological indicators, biomarkers, and imaging parameters has been developed, which holds promise for facilitating tailored clinical decision-making. This model requires further validation and application in larger cohorts.

## Data Availability

The original contributions presented in the study are included in the article/supplementary material. Further inquiries can be directed to the corresponding author.
